# Adaptive Multi-Node Multiple Input and Multiple Output (MIMO) Transmission for Mobile Wireless Multimedia Sensor Networks

**DOI:** 10.3390/s131013382

**Published:** 2013-10-02

**Authors:** Sunghyun Cho, Ji-Woong Choi, Cheolwoo You

**Affiliations:** 1 Department of Computer Science and Engineering, Hanyang University, 55 Hanyangdaehak-ro, Sangnok-gu, Ansan, Gyeonggi-do 426-791, Korea; E-Mail: chopro@hanyang.ac.kr; 2 Department of Information and Communication Engineering, DGIST, 333 Techno jungang-daero, Hyeonpung-myeon, Dalsung-gun, Deagu 711-873, Korea; E-Mail: jwchoi@dgist.ac.kr; 3 Department of Information and Communications Engineering, Myongji University, 116 Myongji-ro, Cheoin-gu, Yongin, Gyeonggi-do 449-728, Korea

**Keywords:** mobile wireless multimedia sensor networks, multi-node MIMO, diversity, spatial correlation

## Abstract

Mobile wireless multimedia sensor networks (WMSNs), which consist of mobile sink or sensor nodes and use rich sensing information, require much faster and more reliable wireless links than static wireless sensor networks (WSNs). This paper proposes an adaptive multi-node (MN) multiple input and multiple output (MIMO) transmission to improve the transmission reliability and capacity of mobile sink nodes when they experience spatial correlation. Unlike conventional single-node (SN) MIMO transmission, the proposed scheme considers the use of transmission antennas from more than two sensor nodes. To find an optimal antenna set and a MIMO transmission scheme, a MN MIMO channel model is introduced first, followed by derivation of closed-form ergodic capacity expressions with different MIMO transmission schemes, such as space-time transmit diversity coding and spatial multiplexing. The capacity varies according to the antenna correlation and the path gain from multiple sensor nodes. Based on these statistical results, we propose an adaptive MIMO mode and antenna set switching algorithm that maximizes the ergodic capacity of mobile sink nodes. The ergodic capacity of the proposed scheme is compared with conventional SN MIMO schemes, where the gain increases as the antenna correlation and path gain ratio increase.

## 1. Introduction

During the last several decades, wireless sensor networks (WSNs) have been widely used in various areas, including environmental monitoring, home automation, healthcare, agriculture, unmanned battle fields, public space surveillance and intelligent traffic systems (ITSs) [1]. A WSN is usually deployed with static sensor nodes to perform monitoring missions in the region of interest. However, due to dynamic changes in events and hostile environment, a purely static WSN could face severe problems, such as scalability, coverage and unfair battery consumption. To overcome these problems, mobile wireless sensor networks (mobile WSNs) have been proposed [2]. Mobile WSNs are a particular class of WSN in which mobile sink or sensor nodes play a key role in the execution of the applications. Although it has been shown that mobile sensor nodes alleviate several issues related to fixed sensor network coverage and scalability, many technical issues remain [3–8]. One of the most significant challenges for mobile WSNs is to form a reliable wireless channel among a mobile sink node and sensor nodes. Moreover, some mobile WSN applications, such as ITS, unmanned battle fields and public space surveillance, exploit rich sensing information, including video, audio or high definition images. To gather and use rich sensing information, wireless multimedia sensor networks (WMSNs) have been introduced and investigated to determine if sensor nodes are capable of producing different media streams [9,10]. Multimedia information, such as video streams or high definition images, requires much higher data rates than those supported by current WSNs. It is essential to provide fast wireless links among a sink node and sensor nodes for efficient and fast data aggregation in WMSNs. Thus, a reliable and fast transmission technology for mobile sink and sensor nodes is one of the key technical issues to be tackled for mobile WMSNs.

A multiple antenna technique may be an appropriate solution to provide reliable and high data rate channels in WMSNs. Multiple input and multiple output (MIMO) systems can support higher data rates than a single input and single output (SISO) system under the same transmit power budget and bit-error-rate (BER) performance requirements. MIMO communication exploits the spatial components of the wireless channel to improve the capacity and error rate performance of communication systems through spatial diversity or multiplexing. Diversity schemes, such as orthogonal space-time block codes (OSTBC) [11], are used to combat channel fading and provide increased link robustness. Spatial multiplexing (SM) enables the transmission of multiple parallel data streams as a means to enhance system throughput [12]. A MIMO system has been formerly studied as a promising technology for increasing the channel capacity and reliability in cellular or wireless local area network (LAN) systems [13–18]. However, many research results have been proposed in the last few years to make use of MIMO technologies in WSNs [19–22]. In conventional WSNs, the direct application of multi-antenna techniques to sensor networks is impractical, due to their limited physical size and the complexity of a sensor node, which typically can support only a single antenna. For this reason, cooperative MIMO solutions have been proposed as an alternative solution, where SISO sensor nodes cooperate with each other to form a virtual MIMO [23–26]. Recent advances in cost-effective wireless sensor node architectures on the millimeter level have led to designs of multiple antennas for WSNs [27,28]. In these designs, a sensor node can be comprised of a two-element switched antenna array using a single radio frequency front-end. Based on these results, unlike the previous works, where MIMO has been considered for WSNs only in the cooperative virtual MIMO manner, non-cooperative MIMO techniques have been proposed [20–22]. In [21], and a space-time coded cooperative transmission has been proposed. The proposed scheme is a simple non-cooperative space-time technique for single-radio frequency(RF)-switched antenna systems. It significantly reduces both the transmission and the circuit energy consumption. In [22], the combination of MIMO systems with nonlinear detection has been proposed to achieve low power consumption, due to nonlinear modulation and detection, and higher rates, due to spatial multiplexing with multiple antennas. Although previous works provide efficient ways to exploit MIMO in WSNs, they have not considered the mobility of sensor nodes and, consequently, are effective only in fixed WSNs. In mobile WMSNs, the spatial correlation of a wireless channel between mobile sensor nodes can vary frequently, and thus, the gain of a given MIMO scheme can be degraded.

To overcome this problem, we propose an adaptive multi-node MIMO transmission scheme for mobile WMSNs. In an adaptive MIMO transmission, various MIMO schemes are dynamically exploited based on the correlation of the given channels. In cellular systems, a number of adaptive MIMO transmission techniques have been proposed [29–35]. The concept of switching between diversity and multiplexing modes was first proposed in [29] and [30] to improve the bit error rate for a fixed rate transmission based on instantaneous channel state information. A general approach for switching between diversity and multiplexing modes for higher spectral efficiency in broadband channels has been described in [31], where time-frequency selectivity indicators are used to maximize spectral efficiencies for a predetermined target error rate and, yet, reduce overhead. The research in [29,30] and [32] performs spatial mode adaptation using feedback from the receiver derived from the instantaneous channel state. In spatially-correlated channels, however, the spatial modes may be largely determined by the correlation in the channel. Consequently, other research has focused on spatial adaptation based only on the spatial correlation matrices. An advantage of these approaches is that the spatial correlation matrix varies on a slower time scale than the instantaneous channel realizations [33]. Therefore, correlation-based adaptive approaches require much less feedback overhead and incur a small performance penalty relative to instantaneous adaptation. Along these lines, [34] proposes to switch between statistical beamforming, double space-time transmit diversity and spatial multiplexing with linear receivers and a four-antenna system based on ergodic link capacity (the ergodic mutual information assuming a certain transmit configuration). Forenza *et al.* [35] propose to switch between orthogonal space-time block coding, double space-time transmit diversity and spatial multiplexing with linear receivers for a four-antenna system based on ergodic link capacity, as well as coded bit error rate for bit-interleaved coded modulation.

Unfortunately, there are limitations with regards to applying these works directly to mobile WMSNs. Most of all, they have been designed for cellular systems and considered only single-node (SN) MIMO systems, where all the signals for a target mobile station come from its associated base station. However, the signals for a mobile sink node come from its associated multiple sensor nodes in mobile WMSNs. Thus, conventional SN MIMO switching schemes cannot avoid performance degradation when there is spatial correlation among a mobile sink node and associated sensor nodes. To tackle this problem, we propose an adaptive multi-node (MN) MIMO transmission scheme for mobile WMSNs, which performs spatial MIMO mode adaptation and antenna set switching with associated multiple sensor nodes. The following are the principal contributions of this paper. First, we propose a suitable model for multi-node, spatially-correlated channels that includes the different path gains from each sensor node. This allows us to include the difference between the average received signal-to-noise ratio for each sensor and the mobile sink link. Second, we derive the ergodic link capacity of orthogonal space-time block coding and spatial multiplexing with zero-forcing receivers for MN MIMO systems, using the results from [35] as a starting point. The resulting capacity expressions depend not only on the MIMO transmission scheme, antenna correlation and signal-to-noise power ratio as in the SN systems, but also on the path gain and transmit antenna set selected from multiple sensor nodes. Based on these models, spatial MIMO mode adaptation and antenna set switching algorithms are proposed. The important differences in the proposed scheme from the previous SN MIMO adaptations [29–35] are: that (i) it considers statistical antenna subset selection; and (ii) we exploit structure in the spatial correlation matrices, due to the difference in sensor node locations. The proposed scheme is especially suitable for mobile WMSNs, because mode switching decisions are made statistically on a slower timescale, thus requiring less inter-sensor node coordination. To demonstrate the improved spectral efficiency of the proposed MN MIMO, we consider a numerical example with two sensor nodes, where a mobile sink moves from one sensor node to another, called a boundary region, and which is similar to a handoff region in cellular systems. We plot the ergodic link capacity for each spatial transmission mode as a function of distance for a given correlation model. We show improvements in spectral efficiency at the boundary region of as much as 50% *versus* strict SN MIMO processing.

The rest of the paper is organized as follows. In Section 2, we introduce the target MN MIMO system and channel models. In Section 3, we analytically derive the ergodic capacity of MN OSTBC and SM schemes. The proposed MN MIMO switching scheme in mobile WMSNs is presented in Section 4. Finally, we provide simulation results and concluding remarks in Sections 5 and 6, respectively.

## 2. System and Channel Models

### 2.1. Target System

**Figure 1.**
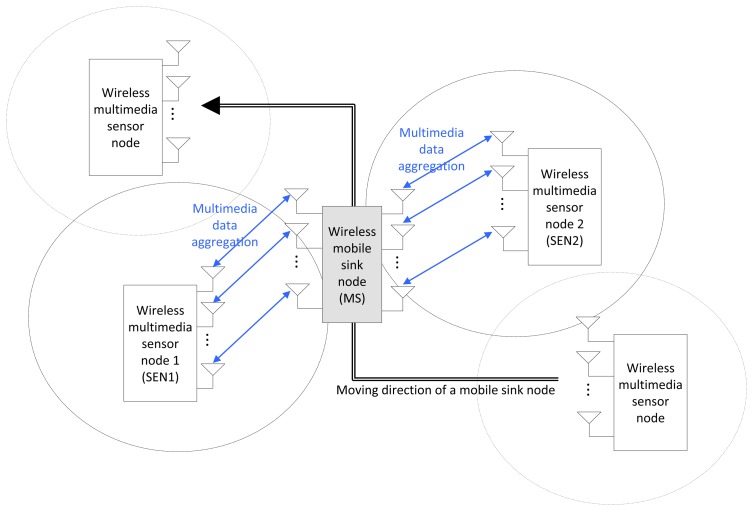
Mobile wireless multimedia sensor networks (WMSNs) with multi-node (MN) multiple input and multiple output (MIMO).

In Figure 1, we illustrate an MN MIMO system model under consideration with two sensor nodes (SENs), each employing *M* or fewer transmit antennas. Unlike the SN MIMO system, any *M* transmit antennas can be selected from 2 *M* antennas of two sensor nodes in the MN MIMO system. An SN MIMO model can be regarded as a subset of general MN MIMO systems when all the signals are sent from a single sensor node (SEN). While we consider two sensor nodes for simplicity of description, the MN MIMO model can be easily extended to cases with more than two sensor nodes. We consider a single mobile sink node (MS) with *N* ≥ *M* and assume that *M* transmit antennas from among the 2 *M* total sensor node antennas will be selected for either spatial multiplexing or diversity transmission. We assume equal transmit power from each sensor node and equal power on each antenna (basically, there is no adaptive power control).

### 2.2. Channel Model for Mobile Wireless Sensor Networks

For purposes of analysis, in this paper, we consider a flat Rayleigh fading channel model with path loss, transmit correlation and receive correlation. The results can be extended to frequency selective fading channels with the use of orthogonal frequency division multiplexing (OFDM). Under these assumptions, the received signal at a target MS can be represented in discrete-time as:

(1) y=Hs+n

where *y* is the (*N* × 1) received signal vector, s is the (*M* × 1) transmitted signal vector, n is the (*N* × 1) independent and identically distributed (i.i.d.) circularly symmetric complex Gaussian noise vector with variance *N*_0_ and **H** = [**h**_1_**h**_2_ …**h***_M_*] is a (*N* × *M*) channel matrix, where **h***_m_* = [*h_m_*_,1_*h_m_*_,2_ …*h_m,N_*]*^T^* is the channel impulse response (CIR) vector from the *m*-th transmit antenna of the assigned SEN to the MS. Here, we omit MS and time indices without loss of generality. Note that the columns of **H** are a function of the transmit antennas and sensor nodes chosen for transmission (this is not explicitly indicated in the notation, but should be apparent in the manuscript).

The channel correlation is assumed to have a Kronecker structure [36]. Further, the channels between different sensor nodes are assumed to be uncorrelated. With these assumptions, the correlation between the CIR's of the *m*_1_-th transmit/*n*_1_-th receive and *m*_2_-th transmit/*n*_2_-th receive antenna pairs can be represented [36] as:

(2) $$E\{h_{{\text{m}}_{1},n_{1}}h_{{\text{m}}_{2},n_{2}}^{*}\}=\{\begin{array}{ll} 0, & \text{different SEN}\\ \sigma_{m}^{2}\eta_{\text{SEN}m,m_{1},m_{2}}\rho_{\text{SEN}m,n_{1},n_{2},} & \text{same SEN}m\;(m=1,2) \end{array}$$

where *E*{} is the expectation operator and *η*_SEN*m,m*_1_*,m*_2__ (*ρ*_SEN*m,n*_1_*,n*_2__) is the transmit (receive) antenna correlation between antenna *m*_1_ and *m*_2_ of SEN*m* (*n*_1_ and *n*_2_ of a target MS), where *η*_SEN*m,m*_1_*,m*_2__ (*ρ*_SEN*m,n*_1_*,n*_2__=1) when *m*_1_ = *m*_2_ (*n*_1_ = *n*_2_). Then, the correlation matrix, Q, of the CIR vector can be decomposed into transmit and received correlation matrices, **R**_*t*_ and **R**_*r*_[36], as:

(3) $$\begin{array}{cc} Q & =\{\text{vec}(H)\text{vec}{\left(H\right)}^{H}\}\\ & =E\left\{\left|\begin{array}{cccc} {\text{h}}_{1}{\text{h}}_{1}^{H} & {\text{h}}_{1}{\text{h}}_{2}^{H} & \cdots & {\text{h}}_{1}{\text{h}}_{M}^{H}\\ {\left({\text{h}}_{1}{\text{h}}_{2}^{H}\right)}^{H} & {\text{h}}_{2}{\text{h}}_{2}^{H} & \cdots & {\text{h}}_{2}{\text{h}}_{M}^{H}\\ \vdots & \vdots & \ddots & \vdots \\ {\left({\text{h}}_{1}{\text{h}}_{M}^{H}\right)}^{H} & {\left({\text{h}}_{2}{\text{h}}_{M}^{H}\right)}^{H} & \cdots & {\text{h}}_{M}{\text{h}}_{M}^{H} \end{array}\right|\right\}\\ & =\left|\begin{array}{cccc} \sigma_{1}^{2} & \sigma_{1,2} & \cdots & \sigma_{1,M}\\ {\left(\sigma_{1,2}\right)}^{*} & \sigma_{2}^{2} & \cdots & \sigma_{2,M}\\ \vdots & \vdots & \ddots & \vdots \\ {\left(\sigma_{1,M}\right)}^{*} & {\left(\sigma_{2,M}\right)}^{*} & \cdots & \sigma_{M}^{2} \end{array}\right|⊗R_{r}\\ & =R_{t}⊗R_{r} \end{array}$$

where *vec*(X) is a vector made by stacking columns of matrix **X**, ⨂ is the Kronecker product and:

(4) $$\begin{array}{l} E\{{\text{h}}_{m}{\text{h}}_{m}^{H}\}=\sigma_{m}^{2}{\text{R}}_{r}\\ E\{{\text{h}}_{m_{i}}{\text{h}}_{m_{j}}^{H}\}=\sigma_{m_{i},m_{j}}{\text{R}}_{r}=\{\begin{array}{ll} 0_{N,N}, & \text{from different SEM}\\ p_{1}^{2}\eta_{\text{SEN}1,m_{i},m_{j}}{\text{R}}_{r}, & \text{from SEN}1\\ p_{2}^{2}\eta_{\text{SEN}2,m_{i},m_{j}}{\text{R}}_{r}, & \text{from SEN}2 \end{array} \end{array}$$

where 0*_A,B_* is the (*A* × *B*) zero matrix and $p_{1}^{2}$ and $p_{2}^{2}$ represent the average power from each sensor node. Note that *p*_1_ and *p*_2_ account for the path gain difference between the two sensor nodes. In most SN MIMO channel models, *p*_1_ = 1. In the MN case, however, the path gain must be retained to account for differences in loss due to different distances between sensor nodes. As a result, the channel matrix of the MN MIMO system can be represented, like the SN MIMO system, as:

(5) $$\text{H}={\text{R}}_{r}^{1/2}{\text{H}}_{w}{\text{R}}_{t}^{T/2}$$

where ${\text{R}}_{r}={\text{R}}_{r}^{1/2}{\text{R}}_{r}^{1/2}$ and ${\text{R}}_{t}={\text{R}}_{t}^{1/2}{\text{R}}_{t}^{1/2}$ are the receive and transmit correlation matrices, and **H***_w_* is an (*N* × *M*) matrix with zero mean, i.i.d. complex Gaussian elements with unit variance.

The SN and MN MIMO channel models have different structures of their transmit correlation matrices, **R***_t_*. For example, when first *M*_1_ and last *M*_2_ antennas are, respectively, selected from SEN1 and SEN2, the transmit channel correlation matrix is:

(6) $$\begin{array}{cc} {\text{R}}_{t} & =\left|\begin{array}{cc} p_{1}^{2}\left|\begin{array}{cccc} 1 & \eta_{\text{SEN}1,1,2} & \cdots & \eta_{\text{SEN}1,1,M_{1}}\\ {\left(\eta_{\text{SEN}1,1,2}\right)}^{*} & 1 & \cdots & \eta_{\text{SEN}1,2,M_{1}}\\ \vdots & \vdots & \ddots & \vdots \\ {\left(\eta_{\text{SEN}1,1,M_{1}}\right)}^{*} & {\left(\eta_{\text{SEN}1,2,M_{1}}\right)}^{*} & \cdots & 1 \end{array}\right| & 0_{M_{1},M_{2}}\\ 0_{M_{2},M_{1}} & p_{2}^{2}\left|\begin{array}{cccc} 1 & \eta_{\text{SEN}2,1,2} & \cdots & \eta_{\text{SEN}2,1,M_{2}}\\ {\left(\eta_{\text{SEN}2,1,2}\right)}^{*} & 1 & \cdots & \eta_{\text{SEN}2,1,M_{2}}\\ \vdots & \vdots & \ddots & \vdots \\ {\left(\eta_{\text{SEN}2,1,M_{2}}\right)}^{*} & {\left(\eta_{\text{SEN}2,1,M_{2}}\right)}^{*} & \cdots & 1 \end{array}\right| \end{array}\right|\\ & =\left|\begin{array}{cc} p_{1}^{2}{\text{R}}_{t,\text{SEN}1} & 0_{M_{1},M_{2}}\\ 0_{M_{2},M_{1}} & p_{2}^{2}{\text{R}}_{t,\text{SEN}2} \end{array}\right| \end{array}$$

where **R***_t_*_,SEN_*_m_* is the transmit antenna correlation matrix of SEN*m* and $\mu (=p_{2}^{2}/p_{1}^{2})$ is the path gain ratio. Assuming $p_{1}^{2}=1$ and $p_{1}^{2}\geq p_{2}^{2}$ without loss of generality, 0 ≤ *µ* ≤ 1. The conventional SN MIMO model with SEN*m* can be represented as a subset of the generalized MN MIMO model as:

(7) $${\text{R}}_{t}=p_{m}^{2}\left|\begin{array}{cccc} 1 & \eta_{\text{SEN}m,1,2} & \cdots & \eta_{\text{SEN}m,1,M}\\ {\left(\eta_{\text{SEN}m,1,2}\right)}^{*} & 1 & \cdots & \eta_{\text{SEN}m,2,M}\\ \vdots & \vdots & \ddots & \vdots \\ {\left(\eta_{\text{SEN}m,1,M}\right)}^{*} & {\left(\eta_{\text{SEN}m,2,M}\right)}^{*} & \cdots & 1 \end{array}\right|.$$

## 3. Link Level Capacity Expressions

This section presents the link level capacity for different candidate MN MIMO transmission algorithms in mobile WMSNs. We use the term link level capacity to denote the ergodic mutual information assuming that equal power allocation is applied at the transmitter [35]. Essentially, we assume that the transmitter is uninformed about the transmit or receive correlation and, thus, does not attempt to perform eigenmode adaptation or water-filling according to the solution of the optimum link level capacity in correlated channels [37]. We assume the perfect knowledge of the CIR at the receiver. The link level capacity under these assumptions for several different SN MIMO transmission algorithms has already been analyzed in [13,14,35,36]. We extend these results to MN MIMO for SM with zero forcing receivers and OSTBC by explicitly accounting for the structure in the MN MIMO transmit correlation matrix in Equation (6).

### 3.1. Spatial Multiplexing (SM)

When SM with zero forcing is employed, the MIMO channel is effectively decoupled into *M* parallel streams, for which the capacity is given by [34]:

(8) $$C_{\text{SM}}=\sum_{k=1}^{M}E[\log_{2}(1+\gamma_{k})]$$

where *γ_k_* is the conditional post-processing signal-to-noise ratio (SNR) for the *k*-th stream. For the zero-forcing (ZF) receiver, *γ_k_* is:

(9) $$\gamma_{k}=\frac{\gamma_{0}}{M}\frac{1}{[{\text{H}}^{H}H^{-1}]k,k}$$

where [**A**]*_m,n_* is an element in the *m*-th row and *n*-th column of matrix **A** and *γ*_0_ = *E_s_*/*N*_0_, where $E_{s}=E\{{\left\|s\right\|}_{2}^{2}\}$. Assuming no receive correlation, *γ_k_* has the probability density function [35]:

(10) $$f(\gamma_{k})=\frac{M\left|{\text{R}}_{t}^{kk}\right|}{\gamma_{0}\left|{\text{R}}_{t}\right|}\frac{\exp\left(-\frac{M_{\gamma k}\left|{\text{R}}_{t}^{kk}\right|}{\gamma_{0}\left|{\text{R}}_{t}\right|}\right)}{(N-M)!}{\left(\frac{M_{\gamma_{k}}\left|{\text{R}}_{t}^{kk}\right|}{\gamma_{0}\left|{\text{R}}_{t}\right|}\right)}^{N-M}$$

where |**A**| is the determinant of matrix **A** and ${\text{R}}_{t}^{kk}$ corresponds to **R***_t_* with the *k*-th row and column removed. Then, the link level capacity can be calculated in a closed form as:

(11) $$C_{\text{SM}}=\sum_{k=1}^{M}\frac{\exp(M|{\text{R}}_{t}^{kk}|/\gamma_{0}|{\text{R}}_{t}|)}{\ln2}\times \sum_{m=1}^{N-M+1}\frac{\Gamma (m-N+M-1,M|{\text{R}}_{t}^{kk}|/\gamma_{0}|{\text{R}}_{t}|)}{{\left(M|{\text{R}}_{t}^{kk}|/\gamma_{0}|{\text{R}}_{t}|\right)}^{m-N+M-1}}$$

where Γ(·,·) is the incomplete gamma function. An upper bound of *C*_SM_ can be written concisely [38] as:

(12) $$C_{\text{SM}}\leq \sum_{k=1}^{M}\log_{2}\left(1+\frac{(N-M+1)|{\text{R}}_{t}|\gamma_{0}}{M|{\text{R}}_{t}^{kk}|}\right).$$

### 3.2. Orthogonal Space-Time Block Coding (OSTBC)

With *M*_1_ and (*M* — *M*_1_) transmit antennas from SEN1 and SEN2, respectively, the link level capacity of OSTBC can be expressed as [35]:

(13) $$C_{\text{OSTBC}}=R_{C}E\left\{\log_{2}\left(1+\frac{\gamma_{0}}{R_{C}M}{\left\|\text{H}\right\|}^{2}\right)\right\}$$

where *R_C_*(= *n_s_*/*N_t_*) is the rate of the OSTBC, *n_s_* is the number of symbols transmitted per block and *N_t_* is the number of symbol periods per block. Using a general result from [35], the probability density function of *η* is found to be:

(14) $$f(\eta )=\sum_{i=1}^{r}\left(\prod_{j=1,j\neq i}^{r}\frac{\lambda_{q,i}}{\lambda_{q,i}-\lambda_{q,j}}\right)\frac{\exp\left(-\frac{\eta }{2\lambda_{q,i}}\right)}{2\lambda_{q,i}}$$

where λ*_q,i_* is the *i*-th eigenvalue of the spatial channel correlation matrix **Q** = **R**_*t*_ ⨂ **R**_*r*_. Using Equations (13) and (14), the capacity is now given by:

(15) $$\begin{array}{ll} C_{\text{OSTBC}} & =R_{C}\overset{r}{\underset{i=1}{\text{∑}}}\overset{r}{\underset{j=1,j\neq i}{\text{∏}}}\left(\frac{\lambda_{q,i}}{\lambda_{q,i}-\lambda_{q,j}}\right)\times \int_{0}^{\infty }\log_{2}\left(1+\frac{\gamma }{2R_{C}N_{t}}\eta \right)\frac{\exp\left(-\frac{\eta }{2\lambda_{q,i}}\right)}{2\lambda_{q,i}}d\eta \\ & =-\frac{R_{C}}{\ln2}\overset{r}{\underset{i=1}{\text{∑}}}\left(\overset{r}{\underset{j=1,j\neq i}{\text{∏}}}\left(\frac{\lambda_{q,i}}{\lambda_{q,i}-\lambda_{q,j}}\right)\times \exp\left(\frac{R_{C}M}{\gamma_{0}\lambda_{q,i}}\right)Ei\left(-\frac{R_{C}M}{\gamma_{0}\lambda_{q,i}}\right)\right) \end{array}$$

where *Ei*(·) is the exponential integral function. The upper bound of OSTBC can be calculated by Jensen's inequality as follows [35]:

(16) $$\begin{array}{ll} C_{\text{OSTBC}} & \leq R_{C}\log_{2}\left(1+\frac{\gamma_{0}}{R_{C}M}E\{{\left\|\text{H}\right\|}^{2}\}\right)\\ & =R_{C}\log_{2}\left(1+\frac{\gamma_{0}N}{R_{C}}\frac{M_{1}+\mu (M-M_{1})}{M}\right)\\ & \leq R_{C}\log_{2}\left(1+\frac{\gamma_{0}N}{R_{C}}\right):\text{SN OSTBC}. \end{array}$$

Note that the capacity of multi-node (MN) OSTBC is slightly lower than that of single-node (SN) OSTBC, even for high transmit antenna correlation, because the ergodic capacity of OSTBC is largely affected not by spatial correlation, but by path gain and the number of transmit antennas, as shown in Equations (13) and (16). Thus, when 0 ≤ *μ* ≤ 1, SN OSTBC always provides better capacity than MN OSTBC, irrespective of the transmit spatial correlation. That is, MN OSTBC is unlikely to be considered in MN MIMO mode switching or antenna subset selection.

## 4. Statistical Multi-Node MIMO Switching in Mobile WMSNs

In this section, we describe the proposed statistical, multi-node, MIMO mode switching algorithm. We use the term statistical because the mode decision is based on the transmit correlation information and the path gains contained in the transmit correlation matrix. Thus, the link level capacity derived in the previous section can be used as a performance measure, assuming coding and interleaving over a large number of channel realizations. Statistical-based mode switching is effective at the boundary region among different sensor nodes.

### 4.1. Motivation

**Figure 2.**
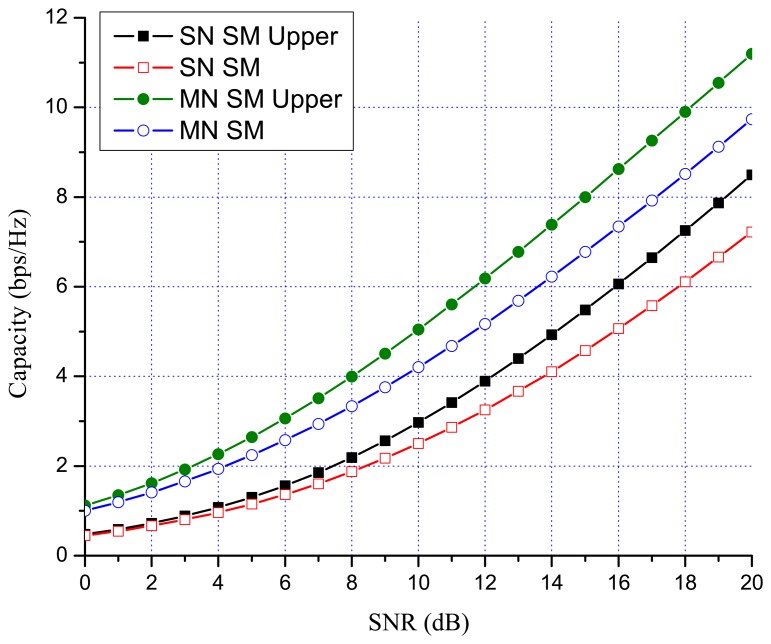
Spatial multiplexing (SM) 2 × 2 MIMO capacity (*η* = 0.8 and *μ* = 0.9).

**Figure 3.**
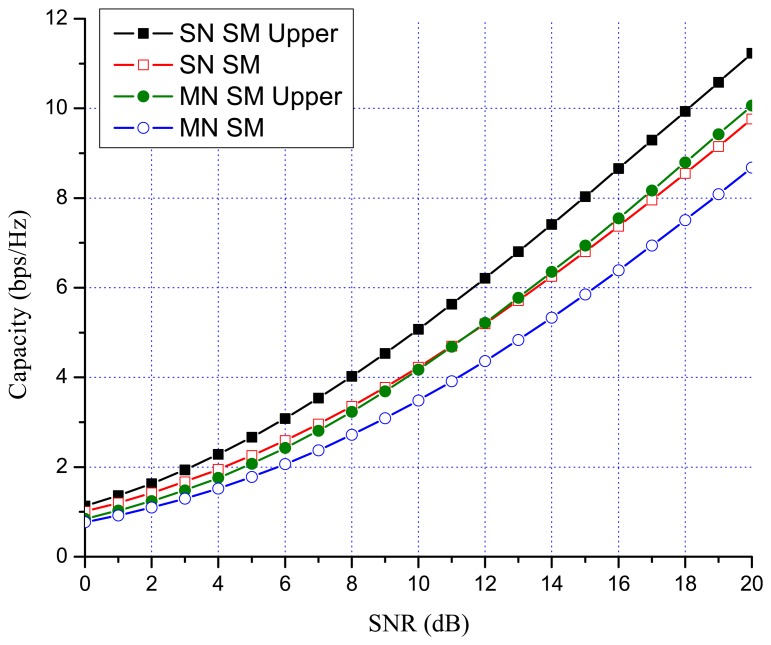
SM 2 × 2 MIMO capacity (*η* = 0.2 and *μ* = 0.4).

**Figure 4.**
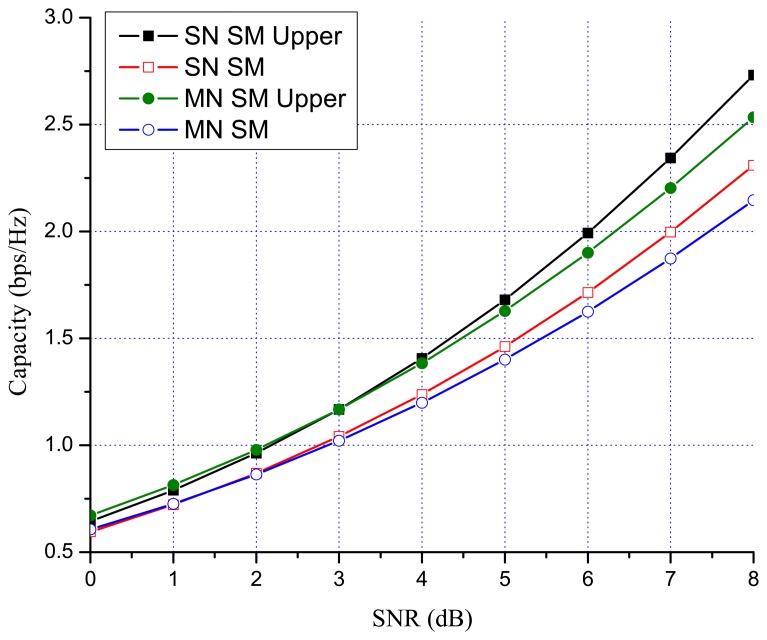
SM 2 × 2 MIMO capacity ( $\eta =1/\sqrt{2}$ and *µ* = 0.125).

Previous works showed that there are many benefits to switching between multiplexing and diversity modes of operation based on the current channel state [30,35,36]. When fast switching is not available, it is reasonable to switch based on the statistics, such as the transmit correlation matrices [38]. In MN MIMO, there are four MIMO possibilities: SN SM, MN SM, SN OSTBC and MN OSTBC. Excluding MN OSTBC, as described in the previous section, SN SM, MN SM and SN OSTBC can be selected in MN MIMO mode switching according to channel environment. In MN MIMO, a capacity crossing can exist between SN SM and MN SM, as well as between SM and OSTBC. To justify this, consider the examples in Figures 2, 3 and 4, which show the link level capacity of 2 × 2 SN SM and MN SM when there is no spatial correlation at the receive antennas (*ρ* = 0). In Figure 2, we assume that the spatial correlation of transmission antennas, *ρ*, is 0.8 and the path gain ratio, *μ*, is 0.9. In this case, MN SM has significantly better performance than SN SM. In Figure 3, we plot the capacity for low spatial correlation (*η* = 0.2) and low path gain ratio (*μ* = 0.4), where SN SM shows higher capacity than MN SM. As shown in Figure 4, when *η* is $1/\sqrt{2}$ and *μ* is 0.125, the upper bound crossing and the exact analysis crossing between SN and MN SM occur at SNR = 3 dB (*γ_MN-SN_* = 2) and around 2 dB, respectively. These results show that MN SM can provide higher capacity than SN SM based on the spatial correlation and path gain ratio. Therefore, adaptive switching between SN and MN MIMO schemes can yield significant capacity gains over SN MIMO switching schemes.

Simple switching between SN and MN modes of operation can be improved further using the concept of antenna subset selection [39]. For example, let us consider the 4 × 4 MIMO case, where more than two correlated transmit antennas may be chosen from one or both sensor nodes. In this case, creating a diagonal transmit correlation matrix is no longer possible, even in MN MIMO schemes, because at least two or more antennas should be chosen from the same SEN. As expected from Equation (11), the lower the correlation among the selected antennas, the higher the capacity of MN SM is. Therefore, adaptive antenna selection according to channel conditions is also required to maximize the MN MIMO capacity.

### 4.2. Algorithm Description

In this section, we propose a statistical MN MIMO mode switching algorithm based on the capacity analysis described in Section 3. The basic idea of the proposed algorithm is the switching of MIMO mode based on the statistical channel quality to maximize the capacity. To find an appropriate switching point and an optimal MIMO mode, we define a multi-node (1) channel estimation, (2) MIMO mode and antenna set selection and (3) MIMO mode switching.

**Figure 5.**
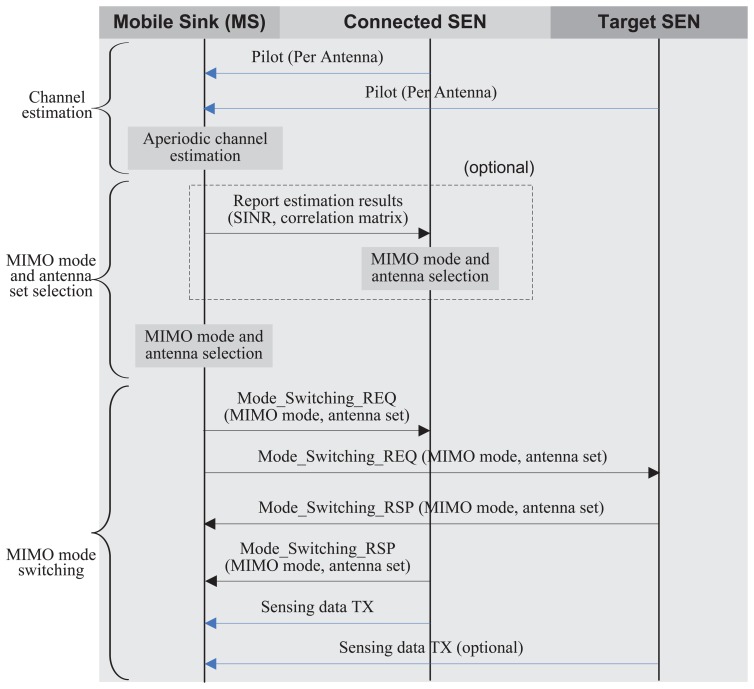
The procedure of a statistical multi-node MIMO switching.

Figure 5 illustrates the overall sequences of the proposed scheme in an MS and SENs. As shown in Figure 5, an MS periodically measures the link quality, including the average SNR and the spatial correlation for a part or all antenna sets from multiple SENs. To measure the link quality, the MS usually exploits the predetermined reference pilot signals that are orthogonally transmitted from transmit antennas [40]. By correlating the received pilot signals transmitted from different antennas for much longer than the channel coherence time, the transmit antenna correlation can be estimated, whereas the receive antenna correlation can be obtained by correlating received pilot symbols of different receive antennas transmitted from the same transmit antenna [41]. The average SNR can also be estimated without much complexity. Based on the channel estimation results, either an SEN or MS determines an appropriate antenna set and a MIMO mode. In the proposed algorithm, ergodic capacity expressions Equations (11) and (13) (or their bounds, Equations (12) and (16)) can be the cost functions to find an optimal antenna set and a MIMO transmission mode. If the SEN determines an antenna set and a MIMO mode, the MS should report the channel estimation result to the SEN. Otherwise, the MS selects an optimal antenna set and MIMO mode and sends a MIMO-mode-switching request to the SEN. To maximize the capacity, SN (or MN) SM or SN OSTBC transmission can be selected from Equations (11) and (13) (or their bounds, Equations (12) and (16)) by calculating the determinants of the correlation matrix, e.g., Equations (6) and (7). Specific SNR crossing-points will be presented in the following section with detailed derivation for the two-antenna case. After antenna and MIMO mode selection, the currently connected SEN notifies the selected antenna set and MIMO mode to the MS, and the MS forwards it to the target SEN by transmitting a Mode Switching REQ message only when the multi-node MIMO scheme is selected. If the target SEN has enough power to support the requested MIMO mode for the MS, then it sends a Mode Switching RSP message to the MS. Then, the MS sends a Mode Switching RSP message to the currently connected SEN with the confirmation of the selected MIMO mode and antenna set. Finally, the connected SEN and the target SEN update the MIMO mode and transmit the sensing data using the selected antenna and MIMO mode.

## 5. Performance Evaluation

This section presents the performance evaluation results of the proposed scheme. To demonstrate an application process of the proposed MIMO mode switching algorithm, we describe the special case of *M* = 2 and *N* = 2. In addition, the numerical results of the exact link level capacity are presented to justify the benefit of MN MIMO mode switching on the link level capacity.

### 5.1. The Application of the Proposed Scheme to the Two-Antenna Case

In this case, we can use closed form solutions for the eigenvalues of 2 × 2 matrices to simplify the link level capacity expressions in the previous section. In this case, the simpler form of link level capacity of Equation (12) is described as:

(17) $$C_{\text{SM}}\leq \sum_{k=1}^{M}\log_{2}\left(1+\frac{\left|{\text{R}}_{t}\right|\gamma_{0}}{2}\left(\frac{1}{\left|{\text{R}}_{t}^{11}\right|}+\frac{1}{\left|{\text{R}}_{t}^{22}\right|}\right)+\frac{{\left|{\text{R}}_{t}\right|}^{2}\gamma_{0}^{2}}{4\left|{\text{R}}_{t}^{11}\right|\left|{\text{R}}_{t}^{22}\right|}\right)$$

where:

(18) $${\text{R}}_{t}=\{\begin{array}{ll} p_{m}^{2}\left|\begin{array}{cc} 1 & \eta_{\text{SEM}m,1,2}\\ {\left(\eta_{\text{SEN}m,1,2}\right)}^{*} & 1 \end{array}\right|, & \text{single}-\text{node}(\text{SEN}m)\\ \left|\begin{array}{cc} p_{1}^{2} & 0\\ 0 & p_{2}^{2} \end{array}\right|, & \text{multi}-\text{node}. \end{array}$$

Note that correlation and path gain are the main parameters that determine the SM capacity. MN transmission can avoid spatial correlation, increasing the capacity, while power loss is experienced due to $p_{2}^{2}(\leq 1)$, decreasing the capacity. Thus, the SM comparison result is significantly dependent upon the path gain ratio and spatial correlation. For the capacity of SN SM, SEN1 SM provides better performance than SEN2 SM when the following inequality holds, and *vice versa*.

(19) $$4p_{1}^{2}(1-{\left|\eta_{\text{SEN}1,1,2}\right|}^{2})+\gamma_{0}p_{1}^{4}{\left(1-{\left|\eta_{\text{SEN}1,1,2}\right|}^{2}\right)}^{2}\geq 4p_{2}^{2}(1-{\left|\eta_{\text{SEN}2,1,2}\right|}^{2})+\gamma_{0}p_{2}^{4}{\left(1-{\left|\eta_{\text{SEN}2,1,2}\right|}^{2}\right)}^{2}$$

which reduces to $p_{1}^{2}\geq p_{2}^{2}$ when the spatial correlations are the same, *i.e.*, *η* = |*η*_SEN1,1,2_| = |*η*_sen2,1,2_|, as intuitively expected. Assuming SEN1 SM is better than SEN2 SM without loss of generality, the MN SM provides higher capacity than SEN1 SN SM when:

(20) $$\gamma_{0}p_{1}^{2}({\left(1-\eta^{2}\right)}^{2}p_{1}^{2}-p_{2}^{2})\leq 2(p_{1}^{2}+p_{2}^{2})-4(1-\eta^{2})p_{1}^{2}.$$

This can be simplified using $p_{1}^{2}=1$ and $\alpha (p_{2},\eta )=p_{2}^{2}+2\eta^{2}-1$ as:

(21) $$\gamma_{0}(\eta^{4}-\alpha (p_{2},\eta ))\leq 2\alpha (p_{2},\eta ).$$

Here, channel environments can be classified into three cases as:

(22) $$\begin{array}{l} C1:\alpha (p_{2},\eta )\leq 0\\ C2:\alpha (p_{2},\eta )>\eta^{4}>0\\ C3:\eta^{4}>\alpha (p_{2},\eta )>0. \end{array}$$

**Figure 6.**
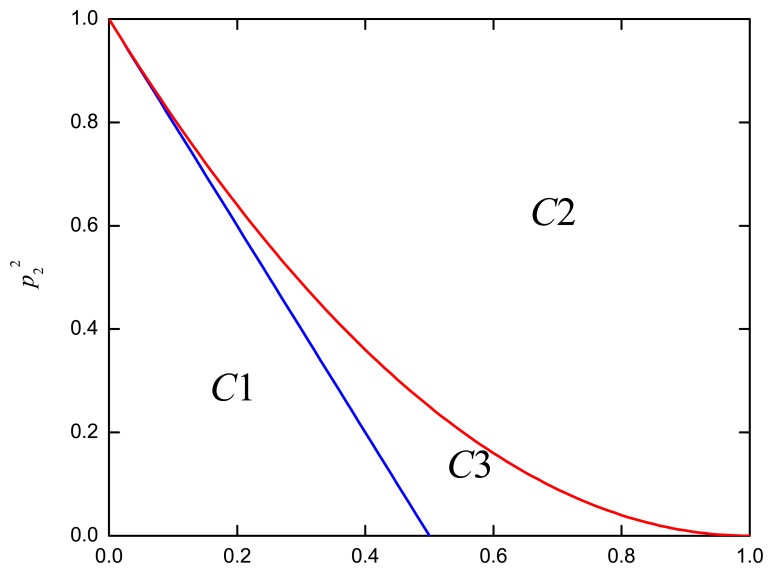
MIMO mode classification for the 2 × 2 case.

The regions for the three cases are depicted in Figure 6. For *C*1, SN SM is always better than MN SM, irrespective of the SNR. This can be intuitively expected, since *α*(*p*_2_, *η*) *≤* 0 corresponds to a smaller *p*_2_ and *η*, where MN processing cannot obtain its advantage over SN MIMO. On the other hand, MN SM is always better than SN SM for *C*2, which corresponds to a major portion in Figure 6. Thirdly, a crossing between SN SM and MN SM occurs in *C*3, where SN SM outperforms MN SM when the SNR is less than the crossing point value, *γ_MN-SN_* (= 2*α* (*p*_2,_ *η*)/(*η*^4^ − *α*(*p*_2,_ *η*))).

We can also calculate SNR crossing points between MN (or SN) SM and SN OSTBC by equating Equations (12) and (16). Although numerical calculation is necessary for a large number of antennas, the SNR crossing point can be easily obtained for two and three antennas. For example, for *M* = *N* = 2, the SNR crossing point, *γ*_OSTBC-SM_, can be represented as:

(23) $$\gamma_{\text{OSTBC}-\text{SM}}=\frac{2\left|{\text{R}}_{t}^{11}\right|\left|{\text{R}}_{t}^{22}\right|}{{\left|{\text{R}}_{t}\right|}^{2}}\left(4-\left|{\text{R}}_{t}\right|\left(\frac{1}{\left|{\text{R}}_{t}^{11}\right|}+\frac{1}{\left|{\text{R}}_{t}^{22}\right|}\right)\right).$$

When SN SM is better than MN SM, *γ*_OSTBC-SM_ = 4(1+ | *η* |^2^)(1− | *η* |^2^)^−2^, whereas γ_ostbc-sm_ = 2*µ*^−2^(3 − *µ*^2^) when MN SM provides higher capacity than SN SM. Therefore, we can select the best MIMO scheme by comparing the capacities for a given SNR and channel condition parameters.

### 5.2. Numerical Results

**Figure 7.**
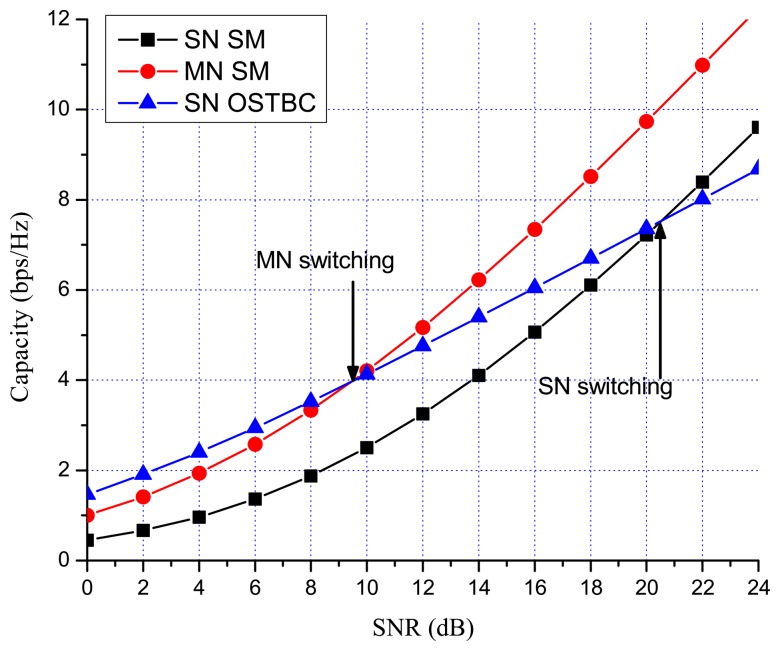
MIMO 2 × 2 capacity with high spatial correlation (*η* = 0.8, *μ* = 0.9).

**Figure 8.**
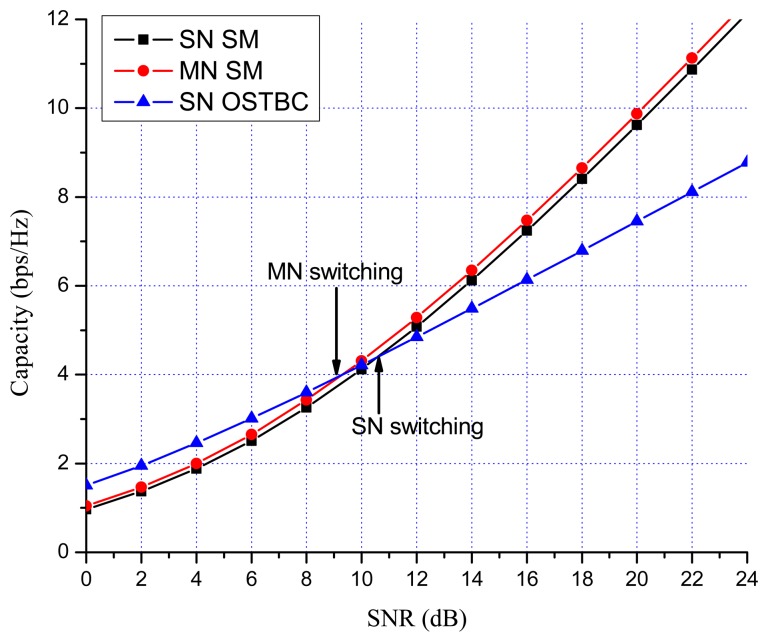
MIMO 2 × 2 capacity with low spatial correlation (*η* = 0.3, *μ* = 0.9).

**Figure 9.**
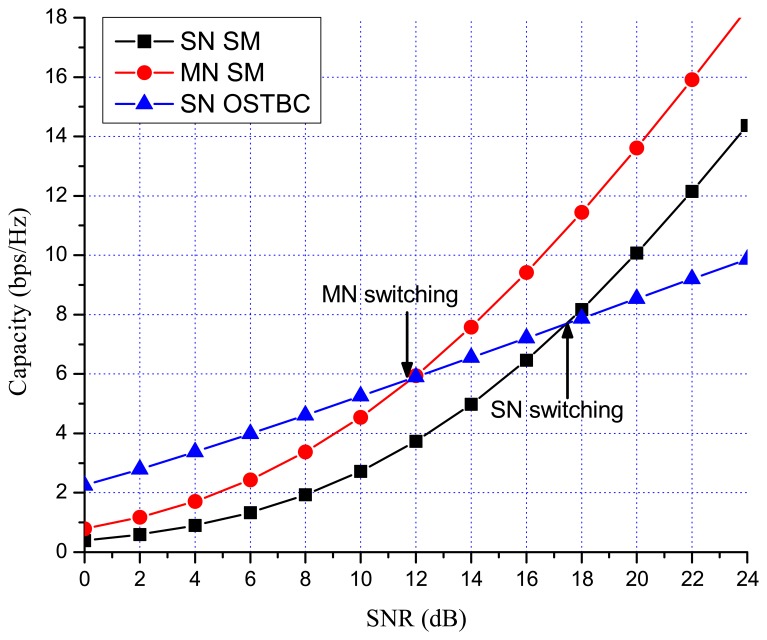
MIMO 4 × 4 capacity with high spatial correlation (*η* = 0.8, *μ* = 0.9).

**Figure 10.**
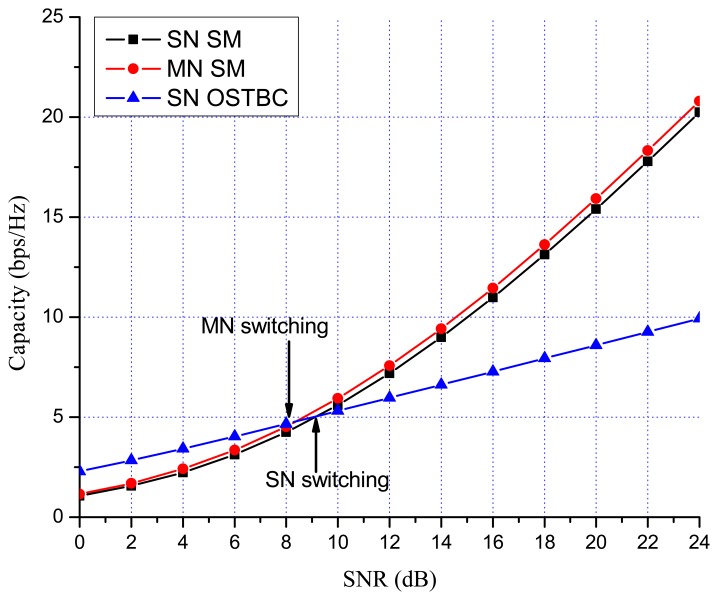
MIMO 4 × 4 capacity with low spatial correlation (*η* = 0.3, *μ* = 0.9).

Figures 7 and 8 show the numerical results of exact link level capacity for *M* = *N* = 2. Figure 7 shows that MN SM provides better capacity than SN SM for *η* = 0.8, regardless of the SNR. Comparing MN SM and SN OSTBC, MN SM outperforms SN OSTBC after the crossing point, γ_OSTBC-SM_, because the capacity of SM increases more rapidly with parallel data transmission [35]. Note that the crossing point decreases as the path gain ratio increases, because MN SM results in better performance for the higher path gain ratio. Figure 8 shows the capacity with low spatial correlation when *η* is 0.3. In this case, the capacities of SN SM and MN SM are almost same, while the SNR crossing point between SN SM and SN OSTBC appears in relatively low SNR region compared to the high spatial correlation case. The SNR crossing points are easily calculated from the upper bound result Equation (23). In Figures 7 and 8, the SNR crossing points, where the MIMO mode switching occurs, are indicated by arrows. Note that the MIMO mode switching points in the proposed scheme are different from the exact analysis obtained from Equations (11) and (13), but the error is marginal, as illustrated in Figures 7 and 8. Figures 9 and 10 show the ergodic capacity of the 4 × 4 case where OSTBC with a code rate (RC) of 3/4 is employed. For MN SM, the antenna set is chosen to maximize the capacity. The performance tendency is similar to the 2 × 2 case; however, the capacity gain of MN SM increases and OSTBC has less benefit, because of the increased number of antennas. These capacity gains can be achieved when a sink node moves slowly. If a sink node moves rapidly and, consequently, the MIMO channel between the sink node and a sensor node changes more shortly than the MIMO mode switching period, then the proposed mode switching scheme may not immediately yield the upper bound plotted in Figures 7, 8, 9, and 10.

## 6. Conclusions

In this paper, we introduced a MN MIMO switching scheme for spatially-correlated channels. The channel for MN systems is modeled first, and then, the ergodic capacities of diversity-based OSTBC and multiplexing-based SM for multiple sensor nodes are derived in closed form. We then proposed a switching scheme, where the MIMO scheme and transmit antenna set are selected to maximize the link level capacity. While the MN OSTBC is less advantageous for MN MIMO irrespective of transmit antenna correlation, MN SM can provide substantial gain over SN SM, especially when the transmit antenna correlation and path gain ratio are relatively high. The proposed MIMO switching scheme can be directly applied to the slow moving sink node when mode switching occurs infrequently. Further studies should extend the scheme to a fast moving sink node, where MIMO mode switching is performed based on instantaneous and average channel condition parameters. In addition, we will study an adaptive MIMO mode switching with consideration for the mobility of wireless sensor nodes.

## References

[1] Yick J., Mukherjee B., Ghosal D. (2008). Wireless sensor network survey. Comput. Netw..

[2] Rezazadeh J., Moradi M., Ismail A.S. (2012). Mobile wireless sensor networks overview. Int. J. Comput. Commun. Netw..

[3] Rezazadeh J., Moradi M., Ismail A.S. Efficient Localization via Middlenode Cooperation in Wireless Sensor Networks.

[4] Meghanathan N. (2010). Impact of the Gauss-Markov mobility model on network connectivity, lifetime, and hop count of routers for mobile Ad-hoc networks. J. Netw..

[5] Amundson I., Koutsoukos X.D. A Survey on Localization for Mobile Wireless Sensor Networks.

[6] Ekici E., Gu Y., Bozdag D. (2006). Mobility-based communication in wireless sensor networks. IEEE Commun. Mag..

[7] Munir S.A., Ren B., Jiao W., Wang B., Xie D., Ma J. Mobile Wireless Sensor Network: Architecture and Enabling Technologies for Ubiquitous Computing.

[8] Amundson I., Koutsoukos X., Sallai J. Mobile Sensor Localization and Navigation Using RF Doppler Shifts.

[9] Misra S., Reisslein M., Xue G. (2008). A survey of multimedia streaming in wireless sensor networks. IEEE Commun. Surv. Tutor.

[10] Akyildiz I.F., Melodia T., Chowdhury K.R. (2008). Wireless multimedia sensor networks: Applications and testbeds. Proc. IEEE..

[11] Tarokh V., Jafarkhani H., Calderbank A.R. (1999). Space-time block codes from orthogonal designs. IEEE Trans. Inform. Theory.

[12] Alamouti S.M. (1998). A simple transmit diversity technique for wireless communications. IEEE J. Sel. Areas Commun..

[13] Blum R.S., Winters J.H., Sollenberger N.R. (2002). On the capacity of cellular systems with MIMO. IEEE Commun. Lett..

[14] Andrews J.G., Choi W., Heath R.W. (2007). Overcoming interference in spatial multiplexing MIMO cellular networks. IEEE Trans. Wirel. Commun..

[15] Fa R., Lamare R.C. (2011). Multiple branch successive interference cancellation for MIMO spatial multiplexing system: Design, analysis and adaptive implementations. IET Commun..

[16] Jiang J., Thompson J.S., Sun H. (2011). A singular-value-based adaptive modulation and cooperation scheme for virtual-MIMO systems. IEEE Trans. Veh. Technol..

[17] Yasir M., Mughal M.J., Gohar N.D., Moiz S.A. Performance Comparison of Wavelet Based OFDM (WOFDM) V-BLAST MIMO Systems with Different Detection Algorithms.

[18] Lee H., Jeon H., Jung H., Lee H. A Novel Detection Algorithm Using the Sorted QR Decomposition Based on Log-Likelihood Ration in V-BLAST Systems.

[19] Jayaweera S.K. (2007). V-BLAST-based virtual MIMO for distributed wireless sensor networks. IEEE Trans. Commun..

[20] Rafique Z., Seet B.C. Energy Efficient Wavelet Based OFDM for V-BLAST MIMO Wireless Sensor Networks.

[21] Tsakalaki E.P., Alradadi O.N., Kalis A., Papadias C.B., Prasad R. (2012). Non cooperative space-time communication for energy efficiency in sensor networks. IEEE Trans. Wirel. Commun..

[22] Psaltopulos G.K., Wittneben A. (2010). Nonlinear MIMO: Affordable MIMO technology for wireless sensor networks. IEEE Trans. Wirel. Commun..

[23] Rafique Z., Seet B.C., Al-Anbuky A. (2013). Performance analysis of cooperative virtual MIMO systems for wireless sensor networks. Sensors.

[24] Cui S., Goldsmith A.J., Bahai A. (2004). Energy-efficiency of MIMO and cooperative MIMO techniques in sensor networks. IEEE J. Sel. Areas Commun..

[25] Li X., Chen M., Lui W. (2005). Application of STBC-encoded cooperative transmissions in wireless sensor networks. IEEE Signal Process. Lett..

[26] Jayaweera S.K. (2006). Virtual MIMO-based cooperative communication for energy-constrained wireless sensor networks. IEEE Trans. Wirel. Commun..

[27] Leang D., Kalis A. Smart Sensor DVB: Sensor Network Development Boards with Smart Antennas.

[28] Kounoudes A., Kalis A., Onoufriou T., Constantinides A.G. Smart Wireless Sensor Technology for Continuous Health Monitoring of Structures.

[29] Heath R.W., Paulraj A.J. Switching between Multiplexing and Diversity Based on Constellation Distance.

[30] Heath R.W., Paulraj A.J. (2005). Switching between diversity and multiplexing in MIMO systems. IEEE Trans. Commun..

[31] Catreux S., Erceg V., Gesbert D., Heath R.W. (2002). Adaptive modulation and MIMO coding for broadband wireless data networks. IEEE Commun. Mag..

[32] Heath R.W., Love D.J. (2005). Multi-mode antenna selection for spatial multiplexing with linear receivers. IEEE Trans. Signal Process..

[33] Herdin M., Bonek E. A MIMO Correlation Matrix Based Metric for Characterizing Non-Stationarity.

[34] Forenza A., McKay M.R., Pandharipande A., Heath R.W., Collings I.B. Capacity Enhancement via Multi-Mode Adaptation in Spatially Correlated MIMO Channels.

[35] Forenza A., McKay M.R., Collings I.B., Heath R.W. Switching between OSTBC and Spatial Multiplexing with Linear Receivers in Spatially Correlated MIMO Channels.

[36] Paulraj A., Nabar R., Gore D. (2003). Introduction to Space-Time Wireless Communications.

[37] Kiessling M., Speidel J., Reinhardt M. Ergodic Capacity of MIMO Channels with Statistical Channel State Information at the Transmitter.

[38] Forenza A., McKay M. R., Pandharipande A., Heath R.W., Collings I.B. (2007). Adaptive MIMO transmission for exploiting the capacity of spatially correlated channels. IEEE Trans. Veh. Technol..

[39] Molisch A.F., Win M.Z. (2004). MIMO systems with antenna selection. IEEE Microw. Mag..

[40] Ma X., Yang L., Giannakis G.B. (2005). Optimal training for MIMO frequency-selective fading channels. IEEE Trans. Wirel. Commun..

[41] Wong T.F., Park B. (2004). Training sequence optimization in MIMO systems with colored interference. IEEE Trans. Commun..

